# Cloning, characterization and expression analysis of a novel gene encoding Kunitz-type protease inhibitor from *Dolichos biflorus*

**DOI:** 10.1007/s13205-012-0047-7

**Published:** 2012-03-24

**Authors:** Kalika Kuhar, Rekha Kansal, Amit Mishra, Kirpa Ram Koundal, Vijay Kumar Gupta

**Affiliations:** 1 Department of Biochemistry, Kurukshetra University, Kurukshetra, Haryana India; 2 National Research Centre on Plant Biotechnology, Indian Agricultural Research Institute (PUSA), New Delhi, India

**Keywords:** *Dolichos biflorus*, Kunitz type, Open reading frame, Polymerase chain reaction, Trypsin inhibitor

## Abstract

This paper reports the presence of a Kunitz-type protease inhibitor (HGPI) gene in *Dolichos biflorus* for the first time. A full-length protease inhibitor gene with complete open reading frame of 669 bp encoding 222 amino acids was cloned from *D. biflorus* using a PCR-based method. BlastN search showed that the HGPI gene shared 100% homology with *Cicer arietinum* trypsin inhibitor mRNA and 97% with *Cajanus cajan* protease inhibitor. The deduced amino acid sequence exhibited homology with Kunitz-type trypsin inhibitor from chickpea, pigeon pea and soybean. The deduced amino acid sequence contained N-terminal signal sequence of 18 amino acids and had a molecular weight of 24 kDa. The phylogenetic tree also showed close relationship with *Cicer arietinum* and *Cajanus cajan*. Southern blotting revealed the presence of only one copy of HGPI gene in the *D. biflorus* genome. Homology modeling was employed to predict secondary structure and 3D-structural models for the protease inhibitor. The gene was found to be constitutively expressed in different tissues as determined by semi-quantitative RT-PCR. The novel HGPI gene has been submitted to the GenBank with Accession No. FN666416. The isolated novel HGPI gene will broaden the pool of plant defense genes and might be an ideal choice for developing transgenic crops resistant to insect pests, as well as pathogens. In addition, it could be used as a probe for selection of insect- and pathogen-resistant genotypes.

## Introduction

Plant genes that protect against herbivory and pathogen attack may be useful for heterologous expression into food and fiber crops (Sardana et al. 1998). Plant protease inhibitors (PIs) have drawn attention as possible transgenes for defense in crops by blocking the growth and development of herbivorous predators through inhibiting digestive enzymes and providing protection against pathogenic fungi and bacteria through inhibiting hydrolytic enzymes, which they use to gain entry (Johnson et al. 1989). PIs are of particular interest because they are generally the product of a single gene and inhibit proteolytic enzymes of animal and fungal origin, but rarely of plant origin (Brattsten 1991; Hilder et al. 1993). A large number of plant PI genes have been isolated, cloned and sequenced (Valueva et al. 2008; Zhang et al. 2008). The gene size and coding regions of these inhibitors are small, devoid of introns (Wang et al. 2008) and comprise readily identifiable core region covering the invariant cysteine residues. The potential of PI has been demonstrated by the transfer of these genes from different sources to several plants of economic interest, resulting in transgenic plants more resistant to predation (Lingling et al. 2005; Pujol et al. 2005) and pathogens (Qu et al. 2003; Quilis et al. 2007).

Two major serine PIs, Bowman-Birk and Kunitz family, have been studied extensively in plants. PIs of Bowman-Birk family have molecular weight of 8–10 kDa with seven disulfide bonds and are relatively heat stable, whereas those belonging to the Kunitz family have a molecular weight of 20–25 kDa with two disulfide bonds (Ryan 1990). Variations in specificity, timing and site of expression suggest the possible involvement of PIs in different plant functions (Beuning et al. 1994).

Kunitz trypsin inhibitor genes represent one of several seed protein gene families that are highly regulated during the plant life cycle (Goldberg et al. 1989). Genetic studies indicate that a single gene is responsible for encoding a seed protein with Kunitz trypsin inhibitor activity (Orf and Hymowitz 1979). As a group, plant KTIs have extremely diverse protease targets and thus have negative effects on a broad range of phytophagous pests and pathogens. Some plant KTIs are antimicrobial, presumably via inhibition of microbial proteinases (Kim et al. 2005; Park et al. 2005). Transcript-profiling studies have shown that several KTI genes are among the most strongly induced genes after wounding and herbivory (Major and Constabel 2006).

*Dolichos biflorus* is one of the lesser known, unexploited legume of tropics and subtropics grown under dry land agriculture. Considering that *D. biflorus* growth has been restricted to a few specific areas, it represents a potential source of genes for insect control. There are earlier reports for the presence of Bowman-Birk PIs in *D. biflorus* seeds (Mehta and Simlot 1982; Sreerama et al. 1997). However, protease inhibitor of the Kunitz family from *D. biflorus* has not yet been reported. Sequence analysis of several double headed inhibitors (Odani and Ikenaka 1976) have shown that they consist of two homologous domains, each of which contains a reactive site for a proteinase, suggesting their evolution from a common single-headed ancestor which ultimately gave an indication for the presence of Kunitz-type inhibitor in *D. biflorus*.

In order to broaden the pool of plant defense genes and to identify new and more potent inhibitor proteins for pest control, insect-specific inhibitor proteins and their genes need to be isolated. The PCR method has proven to be a powerful tool for identification of the specific insecticidal genes carried by different leguminous plants (Gao et al. 1998; Ismanizan et al. 2010). Hence, this paper describes the isolation, cloning and characterization of a full-length gene encoding Kunitz-type protease inhibitor from *D. biflorus* using a PCR-based method. The expression of Kunitz-type protease inhibitor gene has also been analyzed in different parts of *D. biflorus* using RT-PCR. The resulting knowledge should provide novel alternatives for the control of different insects and for coping with the problem of resistance.

## Materials and methods

### Materials

Seeds of *Dolichos biflorus* L. were procured from the Department of Plant Breeding and Genetics, HPKVV, Palampur (Himachal Pradesh), India for experimental purposes. PCR reagents, restriction enzymes and Hexalabel^™^ plus DNA labeling kit were purchased from Fermentas Inc., Maryland 21076, USA. QIAquick PCR purification kit and QIAGEN Omniscript Reverse Transcriptase were obtained from QIAGEN, Hilden, Germany, whereas pGEM^®^-T Easy vector kit was from Promega. Thermoscript RT-PCR kit was purchased from Invitrogen Life Technologies California, USA. Primers were commercially synthesized as highly purified salt-free products by Hysel, India. All other reagents were of the finest commercial grade available.

### Extraction of genomic DNA and total RNA

Total genomic DNA was isolated from 8-day-old etiolated seedlings using the CTAB method (Murray and Thompson 1980) followed by RNase treatment. Total RNA was prepared from leaves, roots, stems, pod walls, flowers and 8-day-old etiolated seedlings by LiCl method (Menke et al. 1996) and from freshly harvested seeds by Kansal et al. (2008). The integrity of isolated RNA was qualitatively checked by 1% formaldehyde agarose gel electrophoresis and stained with ethidium bromide (Sambrook and Russel 2001). Isolated total RNA was further used for expression analysis of the PI gene.

### Southern blot analysis

Purified DNA (5 μg) was completely digested with *Eco*RI and *Bam*HI at 37 °C overnight in a total volume of 45 μl and then electrophoresed on 0.8% agarose gel. The restricted sample in agarose gel was blotted onto a Hybond N^+^ membrane (Amersham) according to the manufacturer’s instructions and hybridized with radiolabeled probe of chickpea The TI gene was obtained from NRC on Plant Biotechnology, IARI, New Delhi, India. The probe was prepared by using Hexalabel^TM^ plus DNA labeling kit (MBI, Fermentas) as per the instruction manual. Southern blot was prepared and hybridized using standard procedure (Southern 1975).

### Oligonucleotide PCR primers

Different sets of gene-specific PCR primers for amplification of Kunitz-type PI gene were designed using Primer 3.0 software (http://frodo.wi.mit.edu/primer3/). The primers were selected from highly conserved regions by using a simultaneous alignment tool through computer analysis with Clustal W tool. The primers were designed from the coding region of the genes determined through Softberry software following the standard rules and analyzed using Fast PCR software for self-dimers, melting temperature and priming efficiency. Primer alignment specificity was checked using NCBI blast. The amplified product size was expected to be 700 bp.

### PCR amplification, cloning and sequencing

PCR was carried out to amplify the coding gene of the PI from the isolated genomic DNA with gene-specific primers according to the modified protocol of Sambrook et al. (1989). Standard PCR reaction was performed in a total volume of 25 μl containing 1× PCR buffer, 1.25 mM MgCl_2_, 100 ng of genomic DNA, 2.5 mM dNTP, 1.25 units of Taq DNA polymerase and 1 μM of each oligonucleotide primer. Amplification was carried out with initial denaturation at 94 °C for 3 min followed by 30 cycles of denaturation at 94 °C for 1 min, annealing at 52 °C for 1 min, and extension at 72 °C for 1 min with a final extension phase of 10 min, performed on a Perkin-Elmer Cetus Instruments thermocycler. The amplified PCR product was analyzed by conventional electrophoresis using 1.2% (w/v) agarose gels and ethidium bromide staining (Sambrook and Russel 2001). The PCR product was purified using QIAquick PCR purification kit. According to supplier standard instructions, this purified product was cloned into pGEM^®^-T Easy vector (Promega) and transformed into *Escherichia coli* DH5α competent cells. The transformants were screened by white-blue selection and tested by the method of colony PCR. The colonies showing positive result were sent for sequencing, which was done at TechnoConcept, New Delhi (India). The data obtained has been submitted to EMBL nucleotide database.

### In silico analysis for characterization of full-length PI gene

The vector sequence was recognized in the sequence obtained after sequencing by VecScreen program of NCBI (http://www.ncbi.nlm.nih.gov/VecScreen/VecScreen.html). Nucleotide sequence from genomic clone and its deduced amino acid sequence were identified by the NCBI BLAST program (http://blast.ncbi.nlm.nih.gov/Blast.cgi).

ORF and the number of exons and introns in the sequence were predicted using FGENESH program of Softberry web server on URL (http://linux1.softberry.com/berry.phtml?topic=fgenesh&group=programs&subgroup=gfind). This tool was also used for DNA translation. Restriction map analysis of complete ORF of *D. biflorus* PI sequence was generated using software available at http://www.nebcutter.com.

Multiple sequence alignment for deduced amino acid sequence was done on CLUSTAL W server (Thompson et al. 1994) available at www.genome.ad.jp. Nucleotide composition and the whole protein and atomic composition analysis of protein sequence were done by using BioEdit Software version 7.0.9.1. The computation of various physical and chemical parameters of protein sequence was carried out using the ProtParam package of the ExPASy web server (http://www.expasy.ch/tools/protparam.html). The exact mass of the protein sequence was deduced using the Isotopident package of the ExPASy web server (http://education.expasy.org/studentprojects/isotopident/htdocs/). Prediction of signal peptide sequence was conducted by using SignalP package of the ExPASy web server (http://www.cbs.dtu.dk/services/SignalP/); however, the subcellular localization of the protein was done using TargetP 1.1 tool of the ExPASy web server. The hydrophobic nature of the deduced amino acid sequence was done using ProtScale package of the ExPASy web server (http://www.expasy.ch/tools/protscale.html). The secondary structure of protein was predicted by using PSS Finder package of Softberry web server (http://linux1.softberry.com/berry.phtml?topic=pps&group=programs&subgroup=propt). The three-dimensional structure of the protein was deduced using 3Djigsaw package available on the ExPASy web server (http://bmm.cancerresearchuk.org/~3djigsaw/); however, it was visualized with the help of Rasmol package available at the ExPASy web server. The phylogenetic tree of the deduced amino acid sequence was generated using software available on http://www.123genomics.com/ along with the other PI amino acid sequences collected from GenBank. The data were used to generate a rooted tree by the neighbor joining method.

### Expression analysis of the isolated protease inhibitor gene

The expression of the PI gene encoding Kunitz-type inhibitor protein in the different parts of *D. biflorus* was analyzed by RT-PCR. Total RNA (5 μg) isolated from different tissues was reverse transcribed into cDNA using QIAGEN Omniscript Reverse Transcriptase as recommended by the manufacturer. The synthesized cDNA was further amplified by PI gene-specific primers using optimized PCR conditions as described above, but with 5 μl cDNA instead of 1 μl of genomic DNA and 25 cycles instead of 30 cycles. The amplified PCR product was separated by 1.2% agarose gel electrophoresis.

## Results and discussion

### Southern blotting

**Fig. 1:**
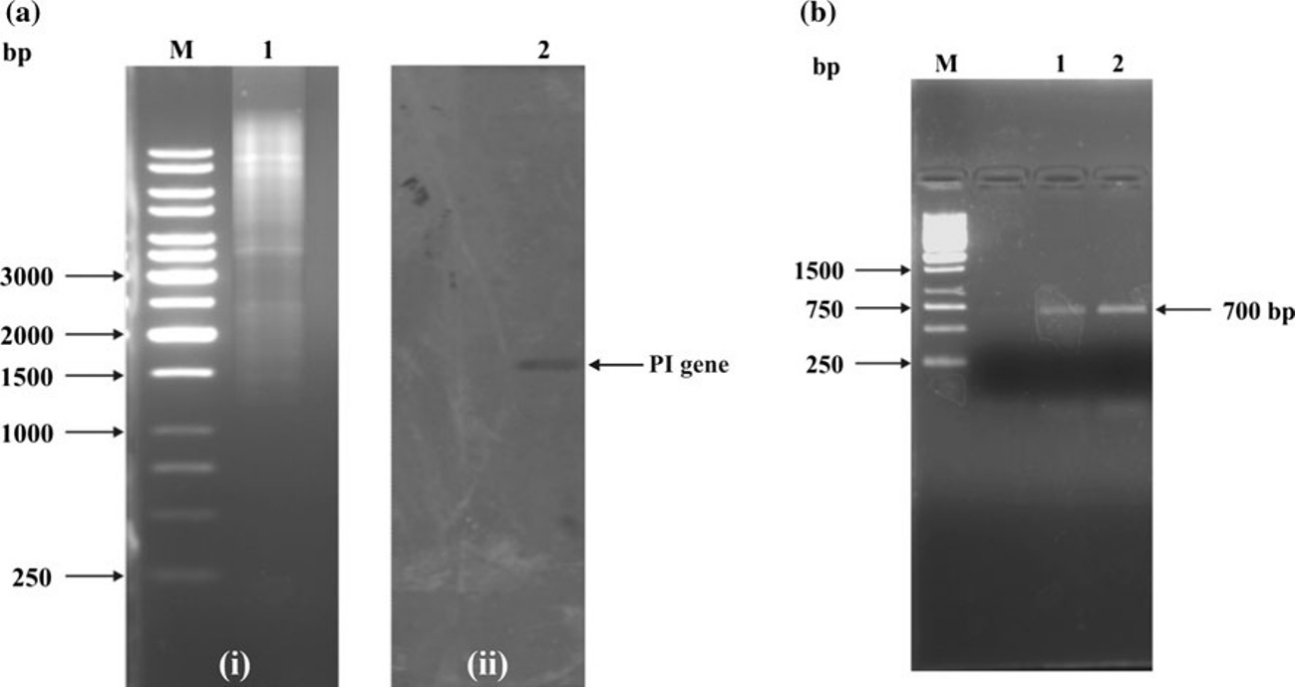
**a** Southern blot hybridization analysis of *Dolichos biflorus* genomic DNA probed with chickpea trypsin inhibitor gene *i* restriction analysis of the genomic DNA digested with *Eco*RI and *Bam*HI. *Lane M* 1 kb ladder, *Lane 1* restricted genomic DNA, *ii* autoradiogram generated from Southern hybridization showing PI gene. **b** PCR amplification of full-length protease inhibitor gene from genomic DNA of *Dolichos biflorus. Lane M* 1 kb ladder, *Lanes 1* and *2* full-length PI gene

The presence of protease inhibitor (PI) gene in *Dolichos biflorus* seedlings was confirmed by Southern blotting. Purified genomic DNA (10 mg) digested with *Eco*RI and *Bam*HI appeared as a continuous smear on 0.8% agarose gel, indicating that the DNA had been restricted. Hybridization of the blot containing restricted DNA with radiolabeled chickpea TI probe revealed the presence of a DNA band of 1.5 kb (Fig. 1a), implying the presence of PI gene in *D. biflorus* seedlings. This observation suggested a considerable homology between chickpea and *D. biflorus* PI genes. Yoshiyuki et al. (2000) observed a single 2.5 kbp fragment of PI gene in Southern blot analysis of soybean when *Eco*RI was used for restriction digestion. Natarajan et al. (2007) also observed single copy gene of Kunitz-type TI in all genotypes of soybean under study by DNA blot analysis.

### PCR amplification

**Fig. 2:**
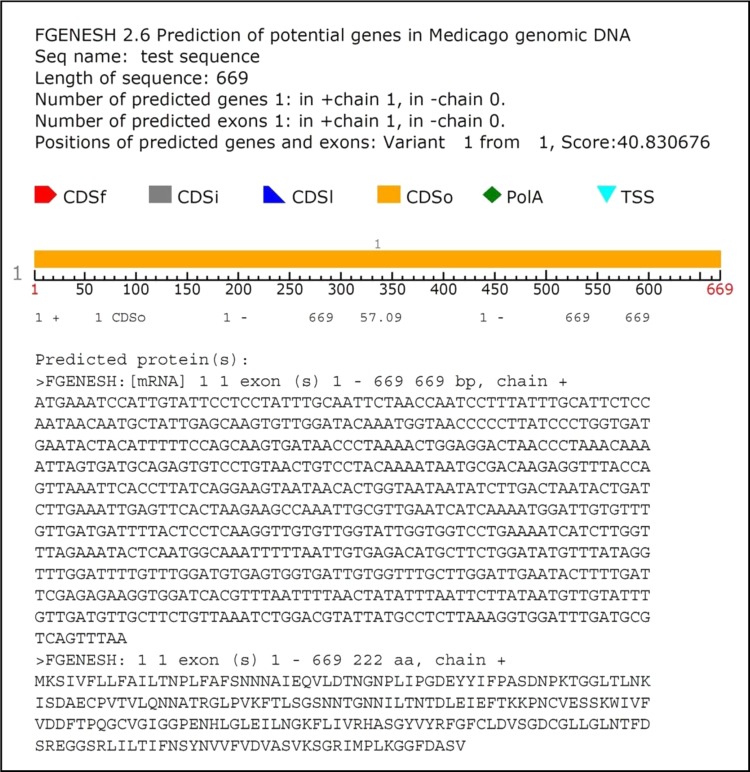
Nucleotide and deduced amino acid sequence of a genomic sequence encoding *Dolichos biflorus* protease inhibitor protein. The start and stop codons (*underlined*) are also shown

The screening of genomic or cDNA library by a DNA probe or antibody is quite time consuming and laborious; the PCR technique was used to directly amplify *D. biflorus* protease inhibitor (HGPI) DNA. PCR amplification reaction was done using the synthetic forward and reverse primers designed from the conserved region of PI gene of legumes. PCR was used to identify regions which were conserved and, therefore, may represent important functional domains. Out of the several primer combinations taken for the PCR, only one with forward primer (5′-ATGAAATCCATTGTATTCTTC-3′) and reverse primer (5′-TTAAACTGACGCATCAAATCC-3′) gave amplification product of the expected size, i.e., ~700 bp when run on 1.2% agarose gel (Fig. 1b). The PCR product was purified, ligated to pGEMT easy vector and transformed successfully. The transformants were selected on LA plates containing X-Gal, ampicillin and IPTG. The white colonies that appeared on the selection plate were employed for plasmid DNA isolation and restricted with *Eco*RI. The restricted samples were found to contain the insert (~700 bp) when run on an agarose gel. Paralleled colony PCR product of 700 bp was amplified with the same primers which coincided with the expected product size, confirming the presence of PI gene in *D. biflorus*. The positive clone was sequenced and the resulting sequence (Fig. 2) was analyzed using bioinformatics tools. Plant PI genes have earlier been isolated through a PCR-based method from various plants including *Apios americana* (Zhang et al. 2008), *Solanum tuberosum* (Valueva et al. 2008) and soybean (Gao et al. 1998).

### In silico analysis of the isolated gene sequence

Internet bioinformatic resources and computer simulation analysis were used to study the DNA sequence. The nucleotide sequence obtained was analyzed on URL http://www.ncbi.nlm.nih.gov. BLASTN search analysis of the sequence on URL http://blast.ncbi.nlm.nih.gov/Blast.cgi showed 100% coverage with *Cicer arietinum* trypsin inhibitor mRNA (Accession No. AJ276262.1). It also showed significant homologies with *Cajanus cajan* protease inhibitor (GU320336.1), *Medicago truncatula* proteinase inhibitor (AF526372.1), *Glycine max* trypsin inhibitor mRNA (EU444603.1) and with other sequences. This indicated that the isolated PI gene belonged to the legume PI gene family.

The PCR fragment encompassed the open reading frame of 669 bp encoding a HGPI precursor with 222 amino acid residues (indicated by one letter code, Fig. 2) as predicted by FGENESH program of Softberry web server. Similar values of open reading frame encoding PI have been reported from other legumes, viz., soybean C-II protease inhibitor gene with 645 bp (Jourdrier et al. 1987), cowpea trypsin inhibitor mRNA with 583 bp (Hilder et al. 1989), cowpea trypsin inhibitor gene with 504 bp (Lawrence et al. 2001) and soybean Kunitz trypsin inhibitor gene with 663 bp (Gao et al. 1998). The sequence did not contain introns. These results were in accordance with the previous reports of intron less PI genes (Wang et al. 2008; Yoshiyuki et al. 2000). In contrast, Ceci et al. (1995) reported that the gene coding for the mustard trypsin inhibitor-2 was discontinuous, being interrupted by a 193-bp long intron.

**Fig. 3:**
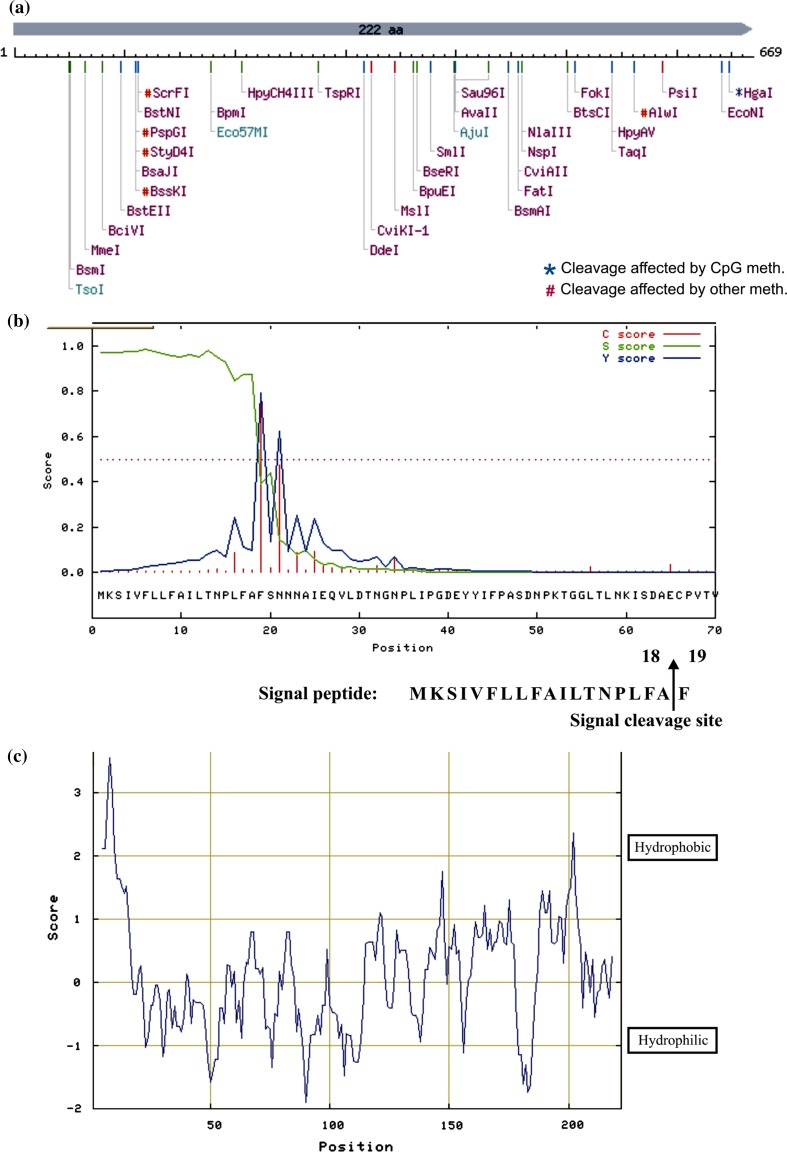
**a** Restriction map of linear sequence encoding *Dolichos biflorus* protease inhibitor. **b** Signal peptide prediction of *Dolichos biflorus* protease inhibitor using ExPASy software. **c** Deduced hydrophobic index of *Dolichos biflorus* protease inhibitor protein using ProtScale package of the ExPASy web server

The deduced MW of the sequence was 4,04,363 Da for double strand with 34.58% (G + C) and 65.42% (A + T) content, which was done using the BioEdit software. Therefore, based on the nucleotide composition, it was clear that the isolated sequence encoding for PI protein was AT rich, similar to that reported by Lawrence et al. (2001) in cowpea trypsin inhibitor mRNA. Restriction map analysis of PI sequence generated using software available at http://www.nebcutter.com indicated that the sequence did not carry restriction sites for the widely used restriction enzymes such as *Bam*HI, *Eco*RI, *Eco*RV, *Hind*III, *Hpa*I, *Nco*I, *Not*I, *Pst*I, *Sac*I, *Sal*I, *Sma*I, *Xba*I, *Xho*I and *Xmn*I (Fig. 3a). To release the PI sequence from the clone, *Eco*RI and *Not*I enzymes were chosen as they were present at the flanking sites at multiple cloning sites (MCS) region and not internally in the ORF.

The deduced *D. biflorus* PI amino acid sequence used as a query sequence in a BlastP search showed the top scoring results with Kunitz proteinase inhibitor (*Cicer arietinum*), protease inhibitor (*Cajanus cajan*), Kunitz trypsin protease inhibitor (*Glycine max*) and so on. This was a very interesting finding of the present study, in that the isolated gene showed homology with Kunitz-type inhibitor, which had not been reported earlier in this crop, although the Bowman-Birk inhibitor gene had already been reported from *D. biflorus* (AM494004).

The protein was found to be rich in glycine, leucine, asparagine, serine and valine as deduced by the BioEdit software (Table 1). A signal peptide of 18 amino acids at the N-terminal end with a cleavage site between amino acid 18 and 19 was presumed by SignalP 3.0 server on the ExPASy web server (Fig. 3b), which were involved in the targeting of proteins to specific compartment as has been reported in many protease inhibitors such as soybean (Hammond et al. 1984), cowpea (Hilder et al. 1989) and pea (Domoney et al. 1995). Since the available evidence from other legumes indicated that PIs were primarily cytosolic proteins, no signals should be necessary. So far, the exact function of signal peptide sequence in the PI could not be assigned (Hilder et al. 1989). Analysis of the predicted *D. biflorus* PI protein sequence by TargetP 1.1 server revealed a secretion pathway score of 0.965, indicating that the inhibitor was likely to be secreted on the outside of the cell.

**Table 1:** Amino acid composition of the *Dolichos biflorus* protease inhibitor protein encoded by the ORF of full-length gene sequence as determined by BioEdit software

Amino acid	Number	Mol %
Ala A	9	4.05
Cys C	5	2.25
Asp D	12	5.41
Glu E	9	4.05
Phe F	16	7.21
Gly G	23	10.36
His H	2	0.90
Ile I	15	6.76
Lys K	10	4.50
Leu L	24	10.81
Met M	2	0.90
Asn N	21	9.46
Pro P	11	4.95
Gln Q	3	1.35
Arg R	6	2.70
Ser S	16	7.21
Thr T	14	6.31
Val V	18	8.11
Trp W	1	0.45
Tyr Y	5	2.25

The whole protein and atomic composition analysis of PI was done by BioEdit Software. ProtParam package of the ExPASy web server computed various physical and chemical parameters of PI protein. The predicted MW of the PI protein was ~24 kDa, of which 21 were negatively charged and 16 were positively charged residues with a theoretical pI of 5.0. This value of MW falls within the range reported previously for Kunitz-type trypsin inhibitor (Lingaraju and Gowda 2008; Oliveira et al. 2007). The total number of atoms present in the protein was 3,374, and the extinction coefficient was 13,200 M^−1^cm^−1^. The instability index depicted a value of 29.45, which suggested the stable nature of this protein. The aliphatic index of this protein was 96.08, while the grand average of hydropathicity was 0.112, confirming the hydrophilic nature of the inhibitor as predicted by the BioEdit software.

**Fig. 4:**
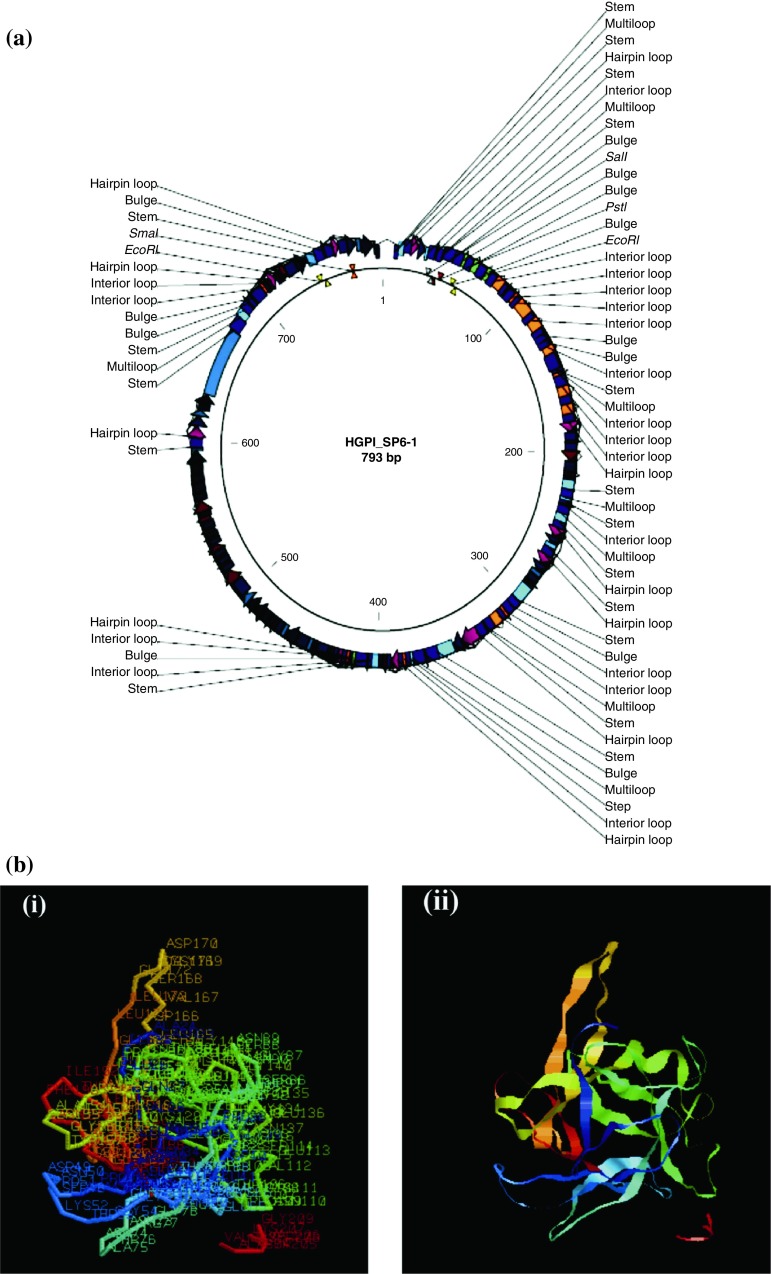
**a** Prediction of secondary structure of *Dolichos biflorus* protease inhibitor protein. **b** Three-dimensional representation of the *Dolichos biflorus* PI using software 3Djigsaw *i* labeled backbone form, *ii* ribbon form

**Fig. 5:**
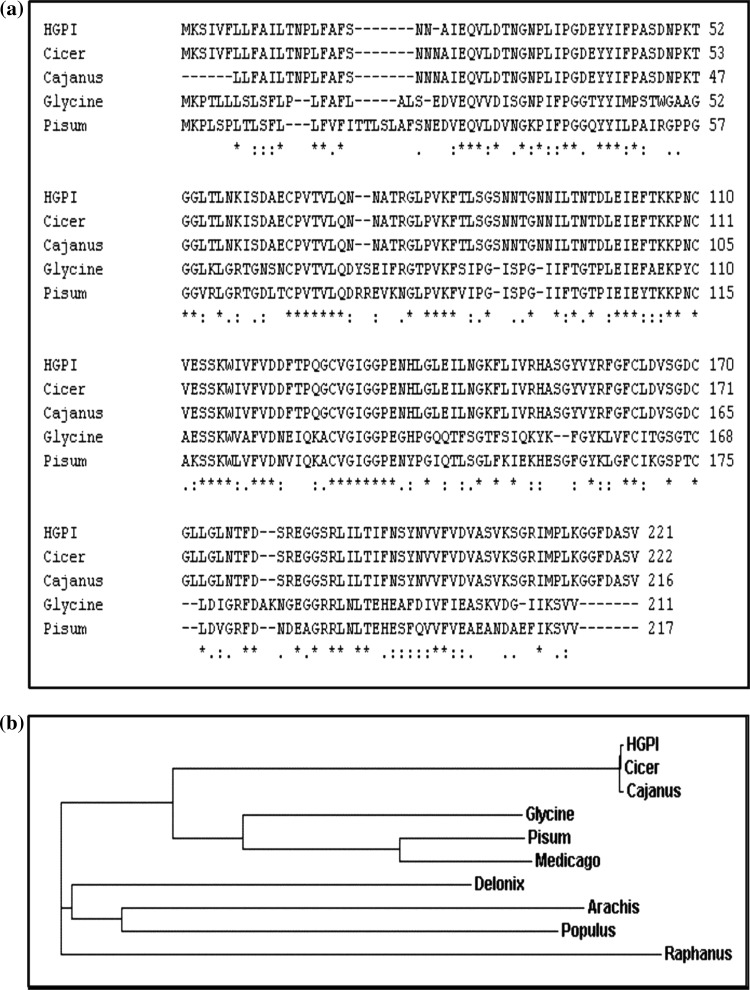
**a** Alignment of deduced amino acid sequence of *Dolichos biflorus* PI gene with other legume PIs CAB76906.1 from *Cicer arietinum*, ADB44827.1 from *Cajanus cajan*, ACA23207.1 from *Glycine max* and O82711.1 from *Pisum sativum*. **b** Phylogenetic tree analysis of *Dolichos biflorus* protease inhibitor (HGPI) with other legume PIs encoding Kunitz-type PIs CAB76906.1 from *Cicer arietinum*, ADB44827.1 from *Cajanus cajan*, AAM88404.1 from *Medicago truncatula*, 1R8N from *Delonix regia*, ACF74332.1 from *Arachis hypogea*, CAI77803.1 from *Populus tremula*, ACC66059.1 from *Raphanus sativus*, O82711.1 from *Pisum sativum* and ACA23207.1 from *Glycine max*

The exact mass of the *D. biflorus* PI was deduced using the Isotopident package of the ExPASy web server. The mono-isotopic mass and the exact mass calculated were 23,981.184 and 23,993.218 Da, respectively. The most likely combination of isotopes was 13.75% and thus the mass of this protein in round figure was 23,981 amu. The hydropathy index deduced using BioEdit Software predicted a hydrophilic nature of this inhibitor and such hydrophilic sequence suggested a cytosolic/extracellular nature of this inhibitor (Fig. 3c). The secondary structure (Fig. 4a) and three-dimensional structure (Fig. 4b) of the *D. biflorus* PI has been predicted. ClustalW analysis using BioEdit software on *D. biflorus* calculated the best match for the *D. biflorus* PI with other PI sequence encoding Kunitz-type trypsin inhibitor available on NCBI gene bank and lined them to identify their similarities and differences (Fig. 5a). A dendrogram displaying phylogenetic relationship showed comparisons of PI sequences encoding Kunitz-type inhibitor in several plants, indicating that TIs from *Cicer arietinum* and *Cajanus cajan* have the closest relationships with *D. biflorus* PI sequence (Fig. 5b). This might be due to the presence of both Kunitz and Bowman-Birk type PIs in these crops. The result was in line with that obtained in BlastP analysis.

### Expression analysis of the isolated protease inhibitor gene

**Fig. 6:**
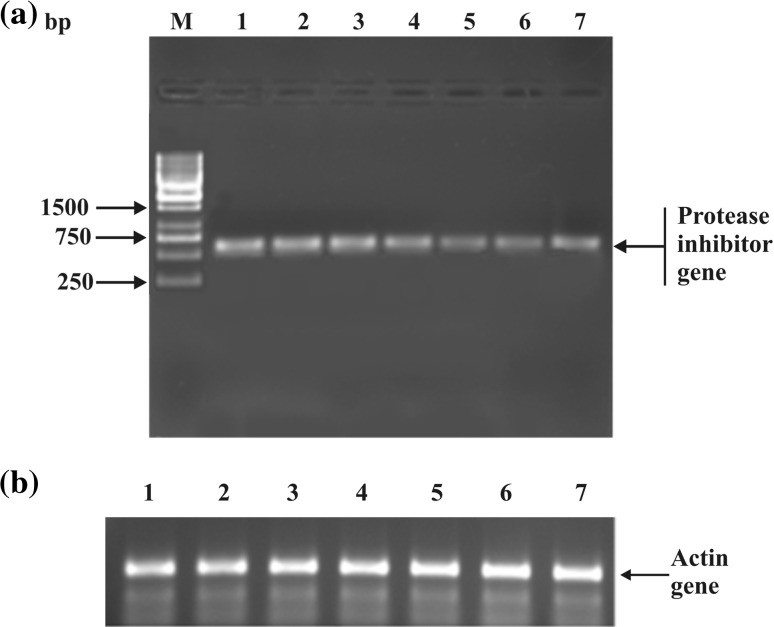
**a** Expression analysis of protease inhibitor in different tissues of *Dolichos biflorus. Lane M* 100 bp DNA ladder, *Lanes 1*–*7* total RNA from *1* seeds, *2* leaves, *3* roots, *4* stems, *5* flowers, *6* pod wall and *7* seedlings. **b** PCR amplification using primers of housekeeping actin gene in different tissues of *Dolichos biflorus. Lanes 1*–*7* total RNA from *1* seeds, *2* leaves, *3* roots, *4* stems, *5* flowers, *6* pod wall and *7* seedlings

The expression of PI was analyzed in different tissues of *D. biflorus* viz., leaves, roots, stems, pod wall, flowers, seedlings and seeds by RT-PCR. The size of the cDNA synthesized ranged from 100 bp to 3 kb, which indicated the intactness of mRNA with minimal degradation. The cDNA reverse transcribed from RNA was subjected to PCR amplification using PI gene-specific primers, which were expected to give an amplicon of ~700 bp. Hence, the presence of ~700 bp amplicon would indicate the expression of PI gene in the tissue. In the present study, 700 bp amplicon was observed in all the tissues viz., leaves, roots, stems, pod wall, flowers, seedlings and seeds of *D. biflorus*, but with differing intensities (Fig. 6a). Simultaneously, parallel PCR was run using primers of housekeeping actin gene as internal control (Fig. 6b). As the PI mRNA was detected in all the tested plant tissues, the *D. biflorus* PI gene was likely to be expressed constitutively. The constitutive expression of PI gene has also been observed by other researchers (Kim et al. 2009; Zhang et al. 2004). The same size of the amplified product from genomic as well as cDNA suggested that *D. biflorus* PI gene may not contain an intron. This was further evident from in silico studies of the PI gene. The physiological functions of PIs were indicated to be mainly based on reports of their developmental and tissue-specific expression patterns (Botella et al. 1996). Expression analysis of this gene will provide fundamental information to facilitate their manipulation as a source of insect resistance in the development of transgenic plants.

## Conclusion

A novel full-length Kunitz-type trypsin inhibitor gene was isolated from *D. biflorus* using a PCR-based method. The gene was further characterized using bioinformatics tools. RT-PCR analysis revealed constitutive expression of the PI gene in different tissues of *D. biflorus*. Based on preliminary experiments in which the *D. biflorus* PI protein was found to exhibit antifeedant and anti-fungal activity, it could be inferred that the gene isolated from this crop could be exploited for generating transgenic plants, effective against insects and pathogen.
